# Structure–Properties Correlations in Novel Copoly(urethane-imide) Films Selectively Destructed Under Thermolysis and Hydrolysis in Alkaline Media

**DOI:** 10.3390/polym17030329

**Published:** 2025-01-25

**Authors:** Andrei L. Didenko, Tatyana E. Sukhanova, Anna S. Nesterova, Gleb V. Vaganov, Viktor K. Lavrentiev, Ilya A. Kabykhno, Natalia A. Grozova, Elena N. Popova, Almaz M. Kamalov, Konstantin S. Polotnyanshchikov, Tatyana S. Anokhina, I. L. Borisov, Vladislav V. Kudryavtsev

**Affiliations:** 1 A.V. Topchiev Institute of Petrochemical Synthesis, Russian Academy of Sciences, 29 Leninsky Prospekt, 119991 Moscow, Russia; vanilin72@yandex.ru (A.L.D.); annest.2107@yandex.ru (A.S.N.); tsanokhina@ips.ac.ru (T.S.A.); kudryav@hq.macro.ru (V.V.K.); 2 Branch of Petersburg Nuclear Physics Institute Named by B.P. Konstantinov of National Research Centre «Kurchatov Institute»—Institute of Macromolecular Compounds, Bolshoy pr. V.O., 31, 199004 St. Petersburg, Russia; tat_sukhanova@bk.ru (T.E.S.); glebvaganov@mail.ru (G.V.V.); lavrentev1949@mail.ru (V.K.L.); popovaen@hq.macro.ru (E.N.P.); spb.kamalov@gmail.com (A.M.K.); kostya.polotn@gmail.com (K.S.P.); 3 School of Advanced Engineering Studies in Digital Engineering, Peter the Great St. Petersburg Polytechnic University, Polytechnicheskaya, 29, 195251 St. Petersburg, Russia; ilya.kobykhno@gmail.com (I.A.K.); grozova_natalia@mail.ru (N.A.G.)

**Keywords:** segmented copoly(urethane-imide)s, thermal degradation, chemical destruction, polyimide membranes, structure, morphology, mechanical and thermal properties

## Abstract

The paper describes changes in the structure, morphology, mechanical and thermal properties of porous film samples of poly(4,4′-oxidiphenylene)pyromellitimide prepared as a result of selective destruction of urethane blocks in copolymers composed of pyromellitimide blocks and polyurethane blocks. The initial samples of the new composition of statistical copoly(urethane-imide)s (CoPUIs) were prepared via polycondensation methods using pyromellitic dianhydride (PMDA), 4,4′-oxidyaniline (ODA), 2,4-toluylenediisocyanate (TDI), as well as polycaprolactone (PCL) and poly(1,6-hexanediol/neopentylglycol-alt-adipic acid) (ALT) as monomers. The molar ratio of imide and polyurethane blocks in CoPUI was 10:1. The initial films were heated up to 170 °C to complete the polycondensation processes, after which they were subjected to thermolysis and hydrolysis. The thermolysis (thermal degradation) of copolymers was carried out by heating the initial samples to temperatures of 300 °C or 350 °C. Then, the thermolized films were subjected to chemical degradation in hydrolytic baths containing an aqueous solution of potassium hydroxide. As a result, urethane blocks were destroyed and removed from the polymer. The resulting products practically did not contain polyurethane links and, in chemical composition, were practically identical to poly(4,4′-oxidiphenylene)pyromellitimide. NMR and IR spectroscopy, atomic force microscopy, X-ray diffraction, thermogravimetric analysis, differential scanning calorimetry and dynamic mechanical analysis and mechanical properties testing were used to determine the differences in the structure and properties of the initial copolymers and targeted products. The effect of the conditions of destructive processes on the structure, morphology and mechanical properties of the obtained porous polyimide films was determined. From a practical point of view, the final porous films are promising as membranes for filtering aggressive amide solvents at high temperatures.

## 1. Introduction

Due to the unique combination of high temperature resistance, mechanical strength and the dielectric properties of polyimides (PI), materials based on them are widely used in engineering, industry and nanotechnology. A special place among PI materials is occupied by porous PI films and coatings, which are used as gas separation, pervaporation and filtration membranes.

One of the ways to prepare nanoporous PI membranes is the bombardment of polymers with heavy ions with high energy, as a result of which channels of degraded material—tracks—appear at the places where ions pass through the films. Track (nuclear) membrane films can have cylindrical through pores (channels) with a diameter of several nanometers to one or two micrometers, which are formed during the subsequent chemical etching of the degraded material in the track sites [1,2,3,4,5,6,7,8,9]. It was shown [4] that upon destruction, the formation of amide groups and intermolecular crosslinking occurs on the inner surface of the tracks. But, the process of manufacturing and developing polyimide track membranes is extremely knowledge-intensive and expensive and requires high material costs.

In recent years, a design concept for polyimide porous materials has been developed that is accessible to chemical technology, in which it is proposed to use thermal degradation processes in copolymers and imides with thermally unstable comonomers, the so-called “sacrificial” comonomers. This concept has been successfully used in the production of porous carbon films based on polyimides [4,5,6]. In this case, the “sacrificial” blocks were polyurethane blocks in the initial segmented copolymers (urethane-imides). According to [10], the thermal degradation of polyurethanes is caused by the occurrence of reverse reactions at elevated temperatures. As a result, urethane bonds are destroyed to form isocyanates and hydroxyl-containing compounds. In the case of polyurethanes formed from arylisocyanates and alcohols, the decomposition process of the urethane bond begins at a temperature of 200 °C.

It should be noted that for the preparation of porous carbon films, the initial films of a multiblock copoly(urethane-imide) were subjected to two-stage thermolysis (pyrolysis) [5] to form in the first stage the imide structure and then in the second stage the carbon structure of the films. In this case, after thermolysis at the first stage at a temperature of 200–300 °C, the polyurethane blocks are destroyed, resulting in the formation of an intermediate porous PI film. At the second stage, at temperatures of 500–700 °C, a porous carbon film is formed as result of the thermolysis (carbonization) of the PI structure. As shown in [6], urethane blocks decompose at a temperature of 200–300 °C, while imide blocks decompose at higher temperatures—500–700 °C. During thermolysis (pyrolysis), macropores are formed in the thermolabilized urethane block, and meso– and micropores are formed in the thermostable imide block.

In the papers [5,6,7,8,9,11], the initial film samples have the structure of multiblock (segmented) copolymers (urethane-imide), in which each repeating unit of the copolymer contains an imide radical and a high-molecular-weight urethane radical [11,12,13,14], and exhibits properties of highly heat-resistant elastomers.

Our research group develops polyimide filter membranes using the processes of selective destruction of urethane blocks in statistical copolymers containing various imide and “sacrificial” polyurethane (for example, polycaprolactone with terminal urethane groups) blocks in a molar ratio of 10:1 [15,16,17,18,19]. We change the chemical composition of the “sacrificial” polyurethane blocks, chemical nature of imide blocks and processing conditions for the optimization of the final properties of the samples. The resulting porous films are chemically similar to the poly-(4,4′-oxydiphenylene) pyromellitimide films or other polyimides obtained by using the standard method of thermal imidization of polyamide acid, but, in this case, the material displays a specific structural organization and morphology. At the same time, besides multiblock (segmented) copolymers, we also synthesize statistical copoly(urethane-imide)s, which differ from segmented samples by the increased content of imide blocks in macromolecules. Statistical copoly(urethane-imide)s exhibit the properties of thermoplastics. Usually, in segmented samples, the molar ratio of imide and urethane blocks is 1:1, but in our case, we prepare statistical samples using the ratio 10:1. With this ratio of stable imide and “sacrificial” urethane blocks, the porous films prepared on the base of statistical copolymers are similar in chemical composition to the corresponding polyimides, but have a specific structural organization and morphology.

In the present work, the combination of NMR, IR spectroscopy, AFM, SEM, X-ray diffraction, TGA, DSC and DMA methods are used for the investigation of novel porous films prepared on the basis of statistical copoly(urethane-imide)s containing rigid blocks of poly-(4,4′-oxydiphenylene) pyromellitimide. The special interest in poly(4,4′-oxidiphenylene)pyromellithimide is due to the fact that it is the most common and affordable polyimide produced in industry, and moreover, its monomeric base and production technology are well developed.

The aim of the work is to identify differences in the structure, morphology, thermal and mechanical properties of porous films prepared under various conditions of thermolysis and subsequent etching in an alkaline solution of the initial films of statistical copoly(urethane-imide)s containing an increased content of rigid blocks of poly(4,4′-oxidiphenylene)pyromellitimide and novel chemical composition of urethane blocks.

## 2. Materials and Methods

### 2.1. Materials

Pyromellitic anhydride (PM) with a melting point (Tm) of ~283–286 °C (Sigma-Aldrich Co. LLC, Burlington, MA, USA), polycaprolactone (PCL2000) with a molecular weight of Mn = 2000 and a melting point (Tm) of ~50 °C (Sigma-Aldrich Co. LLC, Burlington, MA, USA), poly(1,6 hexanediol/neopentyl-glycol-alt-adipic acid) (ALT900) with the molecular weight Mn = 900 and a melting point (Tm) of ~18 °C (Sigma-Aldrich Co. LLC, Burlington, MA, USA), 2,4-toluene diisocyanate (TDI) with melting point (Tm) of ~20–22 °C (Sigma-Aldrich Co. LLC, Burlington, MA, USA;) and 4,4′-oxydianiline (ODA) with a melting point (Tm) of ~188–192 °C (Sigma-Aldrich Co. LLC, Burlington, MA, USA) were used as the initial substances for the synthesis of polymers. *N,N*-dimethylacetamide (DMAc) (Vecton Co. LLC, Saint-Petersburg, Russia) was used as a solvent.

#### Synthesis of an Initial Macromonomer with Urethane Groups in the Chains

Copolymers of (oxydiphenylene)pyromellitimide with polyurethanes with a molar ratio of 10:1 imide and urethane blocks were prepared as objects for the research in the work. The design of the architecture of copolymers is determined by their purpose: to carry out selective thermal degradation of urethane blocks in polymer chains and, as a result of the subsequent etching of the degradation products (in the volume of the polymer), to prepare polymer systems that approach poly(oxydiphenylene)pyromellitimide in their composition. It is known that the thermal decomposition of polyurethanes takes place intensively in the temperature range of 200–300 °C and polyimides in the range of 500–700 °C. In early works [15,16] etching was successfully carried out in acidic hydrolytic media. In this work, etching was carried out in solutions of potassium hydroxide.

The target copolymers were obtained via the joint polycondensation of 4,4′-oxydianiline with two dianhydrides: commercially available pyromellitic anhydride and macromonomeric anhydride of a given structure synthesized for the first time for this work.

The starting materials for the synthesis of macromonomeric dianhydrides were polycaprolactone (PCL2000), poly(1,6-hexanediol/neopentyl-glycol-alt-adipic acid) (ALT900) and a mixture of polycaprolactone and poly(1,6-hexanediol/neopentyl-glycol-alt-adipic acid) in an equimolar ratio. Since the initial polycaprolactone and poly(1,6-hexanediol/neopentyl-glycol-alt-adipic acid) have terminal hydroxyl groups, these substances can be attributed to polymer polyols. Polymer diols were terminated with isocyanate groups using 2,4-toluene diisocyanate, and the resulting intermediate polymers were terminated with anhydride groups using pyromellitic anhydride. As a result of the attempted double termination of polymer polyols, macromeric dianhydrides of a given structure were obtained. The methods of preparing macromonomers and conducting inter-copolycondensation with 4,4′-oxydianiline are presented in our recent works [15,16].

### 2.2. Synthesis Method

The polymers were synthesized according to the known methods described in [15,16,17,18,19,20,21,22]; the process was carried out in one bulb without the isolation of intermediates. The originality of the work consists in varying the synthesis of simple and complex esters (ether and ester), obtaining copolymers based on them with different lengths and compositions of aliphatic and aromatic blocks, and studying the effect of thermolysis and alkaline hydrolysis on these polymer systems.

### 2.3. Synthesis of Initial Macromonomers with Terminal Anhydride Groups and Urethane Groups in the Chain (Macromonomeric Dianhydrides)

Depending on the desired composition of the target product, 5.0 mmol of poly (caprolactone) (the first variant of the desired composition), or 5.0 mmol of poly (1,6-hexanediol/neopentyl-glycol-alternating-adipic acid) (the second variant of the desired composition), as well as a mixtures consisting of 2.5 mmol poly(caprolactone) and 2.5 mmol poly(1,6-hexanediol/neopentyl-glycol-alternating-adipic acid) (the third variant of the desired composition), were loaded into a bulb equipped with a top-drive stirrer and a tube for supplying and discharging argon. After that, 10.0 mmol of 2,4-toluene diisocyanate was added to the bulb. The mixture loaded into the bulb was heated in a thermostatically controlled bath to 80 °C and kept for 1 h, which led to the formation of macromonomers—bisurethanes with terminal isocyanate groups. Then 10.0 mmol of pyromellite anhydride was added to the bulb. The resulting mixture was heated to 180 °C and stirred for 2 h until the resulting melt was homogenized and the release of carbon dioxide bubbles formed during the reaction of anhydride and isocyanate groups ceased. The reaction mixture was cooled to 160 °C and 23 mL of *N,N*-dimethylacetamide was added to it to dissolve the resulting product. The concentration of the solution by using macromonomers was 15, 20 or 25 wt.% in accordance with the desired version of the composition of the macromonomer. Macromonomers were not isolated from the reaction solution.

### 2.4. Synthesis of Copolymers (Oxydiphenylene)pyromellitamide Acid with Polyurethane (Prepolymers of the Target Product)

A total of 55.0 mol of pyromellite anhydride and 55.0 mmol of 4,4′-oxydianiline were loaded into a bulb containing a solution of the selected macromonomer (see Section 2.3), and 50 mL of *N,N*-dimethylacetamide was immediately poured into the bulb. The reaction mass was intensively stirred for 4 h under an argon current at room temperature to undergo the diamine polyacylation reaction. The resulting prepolymer solution in *N,N*-dimethylacetamide with a concentration of ~30 wt.% was passed through a Schott filter, degassed under vacuum and then processed into films. The synthesis is similar to that described in the our previous articles [15,16,17,18,19].

### 2.5. Preparation of Film Samples of Copolymers (Oxydiphenylene)pyromellitimide with Polyurethanes (Target Polymer Products)

**Scheme 1:**
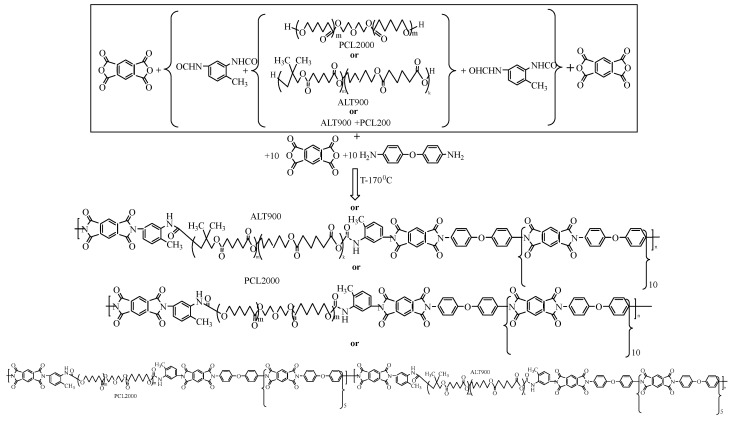
Schematic diagram of the synthesis of the copoly(urethane-imide) samples ODA, PM, TDI, PCL and ALT series. ODA—4,4′-diaminodiphenyl ether; PM—pyromellite anhydride; TDI—2,4-toluene diisocyanate; PCL—poly(caprolactone); ALT—1,6-hexanediol/neopentyl-glycol-alternating-adipic acid.

The obtained prepolymer solutions (see Section 2.4) were cast on hydrophobic glass substrates. Next, the prepolymer films were dried and heated in a thermostat in order to cyclize the amidonic acid units of the prepolymer into the imide units of the target copolymer according to the following regime: for 12 h at 80 °C, 1 h at 100 °C, 1 h at 120 °C, 1 h at 140 °C and finally for 2 h at 170 °C. After that, the films were removed from the plates via immersion in hot water. The thickness of the films was about 100 microns. The stages of CoPUI preparation are illustrated in Scheme 1.

The series of novel copolymers based on the starting substances ODA, PM, TDI, PCL2000 and ALT900 were developed and are listed in Table 1. The final molar ratios of the starting substances in the synthesis of the designated copolymers are also indicated in Table 1.

### 2.6. Thermolysis of Copoly(urethane-imide)s

The copoly(urethane-imide) samples mounted on the glass plates were placed in a SNOL 67/350 programmable thermal oven with natural air convection. The annealing temperature varied from 170 °C to 350 °C with durations ranging from 0.5 h to 2 h. After annealing, the films were removed from the plates (after boiling in water) and examined.

### 2.7. Hydrolysis of Thermolyzed Samples of Copoly(urethane-imide)s

The work combined thermolysis at temperatures of 300–350 °C with the etching of thermolized samples under the conditions of alkaline hydrolysis. Etching was performed by holding the film samples in baths containing 0.04-normal aqueous solutions of potassium hydroxide for a specified time (the duration of hydrolysis was 6, 12, 24, 48 and 72 h). After hydrolysis, the samples were washed with water and dried in air to a constant weight.

### 2.8. Characterization Methods

^1^H NMR spectra of prepolymer solutions were recorded in deuterated DMSO (DMSO-*d_6_*) using a high-resolution AVANCE II-500 WB NMR spectrometer (Bruker, Billerica, MA, USA) at an operating frequency of 500 MHz at room temperature, and the measurement range was 1–10 ppm. ^1^H NMR (in the solid phase) was obtained using the AVANCE III-300 WB device (Bruker, Bruker, Billerica, MA, USA) at an operating frequency of 300 MHz, at room temperature.

FTIR spectra of the CoPUI films were recorded using a Fourier spectrometer “IRAffinity-1S” (Shimadzu, Kyoto, Japan) in the mid-IR region (600–4000 cm^−1^) using a MIRacle microadapter (PIKE Technologies, Madison, WI, USA) with single attenuated total internal reflection.

The characteristic viscosity was measured using the Ubbelode viscometer of the reverse flow of liquids. The viscosity was measured at a temperature of 20 °C in *N,N*-dimethylacetamide (DMAA).

Changes in glass transition and the melting temperature of the CoPUI film samples were determined by using differential scanning calorimetry (DSC) on a DSC 204 F1 (NETZSCH, Zelb, Germany) at a heating rate of 10 °C/min in the temperature range from −60 °C to 300 °C in an inert medium (argon). The weight of the samples was 4–5 mg. As applied to the copolymers under study, the DSC method characterizes the melting processes of the microcrystalline phase formed by aliphatic polyether units [23,24].

Thermogravimetric analysis (TGA) curves were taken using a TG 209 F1 (NETZSCH, Zelb, Germany) in the temperature range from 30 °C to 800 °C at a heating rate of 10 °C/min in an inert medium (argon). The weight of the samples was 2–3 mg.

The temperature dependences of the dynamic values, namely the storage modulus E′, loss modulus E″ and the tangent of the mechanical loss angle (tan δ) of the film samples were obtained via dynamic mechanical analysis (DMA) using a DMA 242 C machine (NETZSCH, Zelb, Germany). Measurements were recorded at a frequency of 1 Hz, a temperature rise rate of 5 °C/min and a deformation amplitude of 0.1%. The glass transition temperatures of the film samples were determined by reference to the temperature of maximum tan δ.

The deformation–strength characteristics of the films were determined at room temperature in the mode of uniaxial tension for the samples in the form of 2 mm wide strips with the length of the working part being 25 mm. The tests were carried out on an Instron 5940 universal tensile testing machine (Instron, Norwood, MA, USA). The tensile testing of the film samples was carried out at a speed of 10 mm/min.

The surface morphology of the synthesized copolymer films was investigated by using the AFM method on the atomic force microscope Nanosurf FlexAFM (produced by Nanosurf AG, Liestal, Switzerland). The shooting was carried out in the dynamic mode using a standard Tap150Al-G cantilever with the resonant frequency of 150 kHz, with a radius of tip curvature of less than 10 nm and cantilever force constant of 5 N/m (produced by Budget Sensor, Sofia, Bulgaria). The overscan was 20%. For each sample, a survey was carried out with a field of 20 μm × 20 μm and 1 μm × 1 μm. For each field of the survey, the results for the *Z*-axis are presented as the topography of the surface in nm and the amplitude, which allows for the better assessing of the topography of the surface at large magnifications. The roughness, coefficient of friction, and friction force in the nano- and microscale of the samples were evaluated. The surface roughness was estimated in scanning areas of 20 μm × 20 μm and 1 μm × 1 μm.

Scanning electron micrographs of the film surfaces as well as film fracture surfaces were obtained using a Supra-55 VP scanning electron microscope (SEM) (Carl Zeiss, Jena, Germany) with a secondary electron detector. The accelerating voltage was 5 kV. The film samples were fixed with a special conductive adhesive on the microscope holders and were sputter coated with a thin layer of platinum under a vacuum.

X-ray studies were performed using an automated X-ray diffractometer DRON-2.0 manufactured by the Leningrad “NPO Burevestnik” (Russia). CuKα-radiation was used. Monochromatization was carried out by using a Ni-filter.

## 3. Results

### 3.1. NMR Spectroscopy Investigations and Characteristic Viscosity of CoPUI Solutions

The probable chemical formulas and designations (conditional composition records) of the synthesized target copolymers are given below:

CoPUI2000PCL

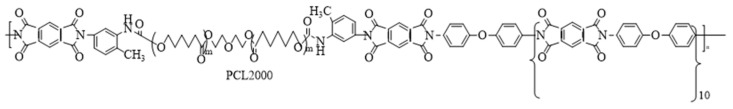


NMR ^1^H spectrum of the prepolymer (DMSO-*d_6_*) δ ppm: 9.96; 8.08; 7.80; 7.60; 7.51; 7.43; 7.30; 7. 17; 7.09; 7.02; 6.80; 4.17; 3.92; 3.60; 2.30; 2.24; 2.18; 1.64; 1.59; 1.30.

The characteristic viscosity of the polymer was [η] = 0.98 × 100 cm^3^ g^−1^ (DMAA, 20 °C).

CoPUIALT900

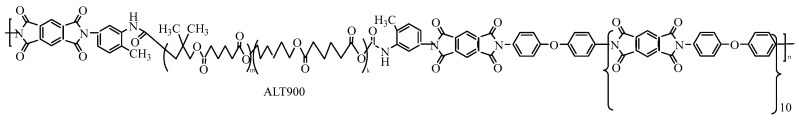


NMR ^1^H spectrum of the prepolymer (DMSO- *d_6_*) δ ppm: 10.1; 8.05; 7.76; 7.54; 7.49; 7.36; 7.29; 7.15; 7.04; 7.00; 6.84; 4.22; 3.98; 3.57; 2.41; 2.29; 1.93; 1.73; 1.67; 1.63; 1.46; 0.88.

The characteristic viscosity of the polymer was [η] = 0.99 × 100 cm^3^ g^−1^ (DMAA, 20 °C).

CoPUIALT900/2000PCL

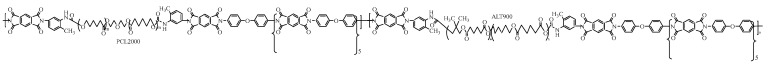


NMR ^1^H spectrum of the prepolymer (DMSO- *d_6_*) δ ppm: 9.92; 8.09; 7.84; 7.61; 7.53; 7.40; 7.22; 7.18; 6.99; 6.90; 6.78; 4.16; 3.96; 3.61; 2.38; 2.30; 2.23; 2.15; 1.87; 1.75; 1.64; 1.56; 1.40; 0.85.

The characteristic viscosity of the polymer was [η] = 0.99 × 100 cm^3^ g^−1^ (DMAA, 20 °C).

### 3.2. NMR Study of CoPUI Films in the Solid Phase

Initial CoPUI2000PCL δ, ppm: 1.25; 1.63; 2.21; 4.03; 7.20; 8.29; 9.90.

After thermolysis (at 300 °C) δ, ppm: 1.27; 1.74; 2.19; 3.08; 5.34; 6.17; 7.11; 8.26.

After alkaline hydrolysis δ, ppm: 1.21; 2.26; 3.13; 5.31; 6.20; 7.20; 8.26.

Initial CoPUIALT900 δ, ppm: 0.89; 1.72; 2.17; 3.92; 7.17; 8.32; 9.96.

After thermolysis (at 300 °C) δ, ppm: 0.93; 1.27; 1.68; 2.10; 3.12; 5.26; 6.20; 7.12; 8.35.

After alkaline hydrolysis δ, ppm: 1.21; 2.12; 3.10; 5.22; 6.34; 7.21; 8.28.

Initial CoPUIALT900/2000PCL δ, ppm: 0.89; 1.20; 1.66; 2.20; 3.99; 7.26; 8.36; 9.89.

After thermolysis (at 300 °C) δ, ppm: 0.89; 1.20; 1.66; 2.20; 3.99; 5.30; 6.19; 7.26; 8.36.

After alkaline hydrolysis δ, ppm: 1.15; 2.08; 3.16; 5.31; 6.25; 7.30; 8.22.

The results obtained indicate the destruction of urethane fragments after thermolysis and alkaline hydrolysis; for example, in the NMR ^1^H spectra of CoPUI films after hydrolysis, there are no signals of protons of urethane NH-groups (in the region of 9.8–10 ppm). However, signals of about 5.30 ppm corresponding to the protons of amino groups, as well as signals of methyl protons (about 1.30 ppm, 2.20 ppm), which are, apparently, products of the destruction of urethane fragments.

The combined table of the NMR study is given in the additional Supplementary Materials of the paper (Table S1).

The obtained results of the NMR-spectroscopic studies are in good agreement with the results of the IR spectroscopic studies of CoPUI films described in Section 3.2.

### 3.3. FTIR-Spectroscopic Study of Synthesized CoPUI Films Before and After Their Selective Destruction

The chemical structure of the prepared multiblock copolymers in the initial state, as well as after thermolysis and subsequent hydrolysis, was identified via IR-spectroscopy.

**Figure 1:**
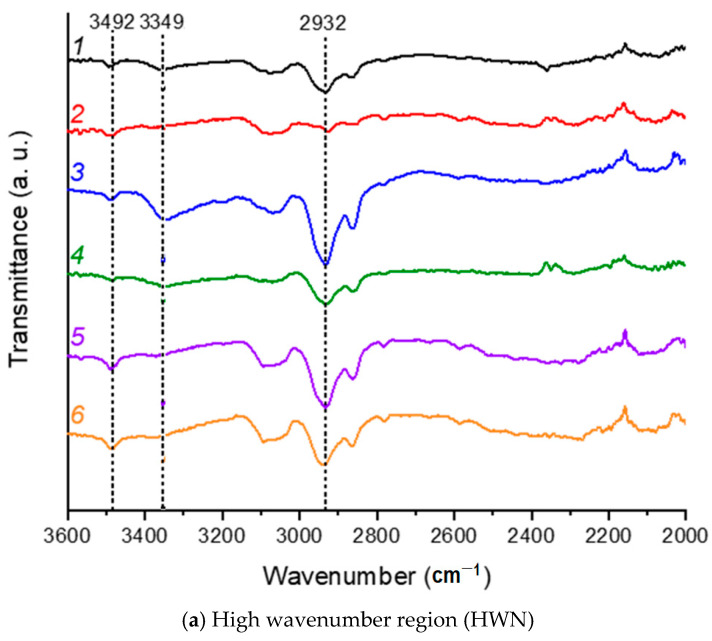

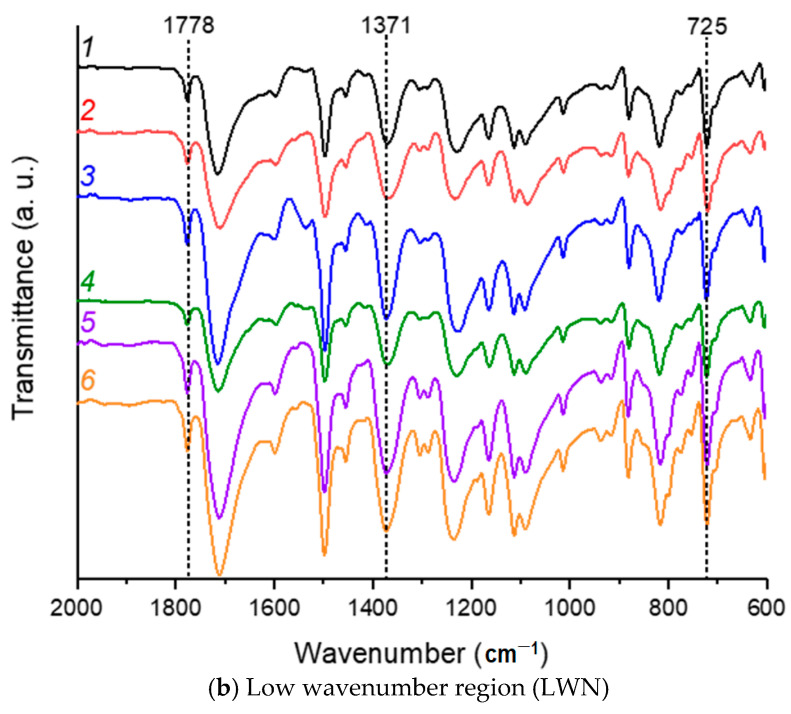
FTIR spectra (**a**)—the range of 3600 up to 2000 wavenumber (cm^−1^) and (**b**)—the range of 2000 up to 600 wavenumber (cm^−1^): 1—CoPUIALT900 heated at 170 °C; 2—CoPUIALT900 thermolized at 350 °C; 3—CoPUIALT900/2000PCL heated at 170 °C; 4—CoPUIALT900/2000PCL heated at 170 °C, then subjected to alkaline hydrolysis in 0.04 N KOH for 2 days; 5—CoPUIALT900/2000PCL thermolized at 350 °C; 6—CoPUIALT900/2000PCL thermolized at 350 °C, then subjected to alkaline hydrolysis in 0.04 N KOH for 2 days.

Figure 1a,b show the FTIR spectra of the CoPUI films: CoPUIALT900—CoPUIALT900 heated at 170 °C (curve 1); CoPUIALT900 thermolized at 350 °C (curve 2); CoPUIALT900/2000PCL—CoPUIALT900/2000PCL heated at 170 °C (curve 3); CoPUIALT900/2000PCL heated at 170 °C, then subjected to alkaline hydrolysis in 0,04 N KOH for 2 days (curve 4); CoPUIALT900/2000PCL thermolized at 350 °C (curve 5); CoPUIALT900/2000PCL thermolized at 350 °C, then subjected to alkaline hydrolysis in 0,04 N KOH for 2 days (curve 6).

These spectra are characterized by the presence of two spectral regions differing in the intensity of the absorption bands: the first region—HWN, 2000–3600cm^−1^; the second region—LWN, 600–2000 cm^−1^. In the first region, there are several very weak absorption bands, and in the second region, there is a series of evenly spaced narrow bands of medium and high intensity.

The presence of aromatic imide rings in polymer chains due to the imidization reaction is characterized by the presence in the spectrum of the CoPUIALT900 sample heated at 170 °C of absorption bands at 1778–1779 cm^−1^—symmetric valence C=O vibrations of imide cycles (imide-I), 1715–1720 cm^−1^, asymmetric stretching C=O vibrations of imide cycles (imide-I), 1371 cm^−1^—symmetric valence C=O oscillations of imide cycles (imide-II), 725 cm^−1^—symmetric valence C=O oscillations of the imide cycles (imide-IV).

The presence of characteristic imide bands in the FTIR spectrum of Figure 1b at 1778 cm^−1^ and 725 cm^−1^ indicates the presence of imide groups in the macromolecules of this CoPUI both before and after thermolysis at 350 °C.

It is known that the values of the wave numbers for the imide-I and imide-II bands depend on the type and molecular weight of the flexible segments [25].

In the FTIR spectrum of the CoPUIALT900 sample heated at 170 °C (Figure 1a), there are also weak bands at 3355 cm^−1^, valence vibrations of N-H bonds in the group –NH_2_ and 2929 cm^−1^ vibrations of aliphatic C-H bonds in the group –CH_2_–. After thermolysis at 350 °C (Figure 1a), the band at 3355 cm^−1^ disappears, and the band at 2929 cm^−1^ shifts towards wave numbers increasing and is recorded at 2932 cm^−1^.

The absence of a peak at 2270 cm^−1^ in the FTIR spectrum of the CoPUIALT900 sample indicates that the NCO groups of macrodiols terminated by 2,4-TDI completely reacted with pyromellitic dianhydride [26].

As follows from numerous literature data [27,28], asymmetric stretching vibrations of non-hydrogen-bonded C=O groups in the urethane fragment usually appear as a band in the region of 1740–1720 cm^−1^ and for bonded ones in the region of 1711–1684 cm^−1^. These bands are absent in the FTIR spectrum of the synthesized CoPUIALT900 sample.

Thus, it is shown that the thermolysis of CoPUI films leads to the degradation (destruction) of PU blocks, while the urethane group decomposes.

Figure 1 also shows the FTIR spectra of a series of CoPUIALT900/2000PCL samples obtained under different conditions: heated at 170 °C (curve 3), thermolized at 350 °C (curve 5), as well as heated at 170 °C, then subjected to alkaline hydrolysis (curve 4) and thermolized at 350 °C, and then subjected to alkaline hydrolysis (curve 6). The analysis of the spectra shows that after thermolysis and hydrolysis, there are no absorption bands in the region of 3300 cm^−1^ corresponding to the fluctuations of N-H urethane groups, and the intensity of the C-H bands of vibrations decreases significantly.

On the contrary, in the spectra of these samples, low-intensity absorption bands in the region of 3340–3350 cm^−1^ are detected, corresponding to the urethane N-H stretching vibrations.

### 3.4. AFM Investigation of the Morphology of CoPUI Films

The surface of the synthesized copoly(urethane-imide) (CoPUI) films was studied via atomic force microscopy (AFM) using scanning matrices 20 × 20 µm (microlevel) and 1 × 1 µm (nanolevel).

Figure S1 in the Supplementary Materials presents AFM images of fragments of the upper (free) and lower (to the glass substrate) surfaces of the copolymer CoPUIALT900 film heated to 170 °C, in the amplitude mode (scanning matrix 20 × 20 µm). The AFM images (Figure S1(1A,B)) show numerous lines and grooves extending along the entire surface of the sample, which intersect, as a rule, at an angle of 30°. These grooves were formed, apparently, on the upper surface because of the impact of a squeegee blade used in forming the film, and on the lower surface because of contact with scratches on the glass substrate. Irregularly shaped particles ranging in size from 300 to 500 nm are located along the grooves. This leads to the formation of a rather rough relief of both surfaces of the film, so the parameters of the arithmetic mean and RMS roughness of this sample are quite large and amount to Ra = 167.7 nm and Rq = 194.7 nm (for the upper surface) and Ra = 27.3 nm and Rq = 31.9 nm (for the lower surface).

**Figure 2:**
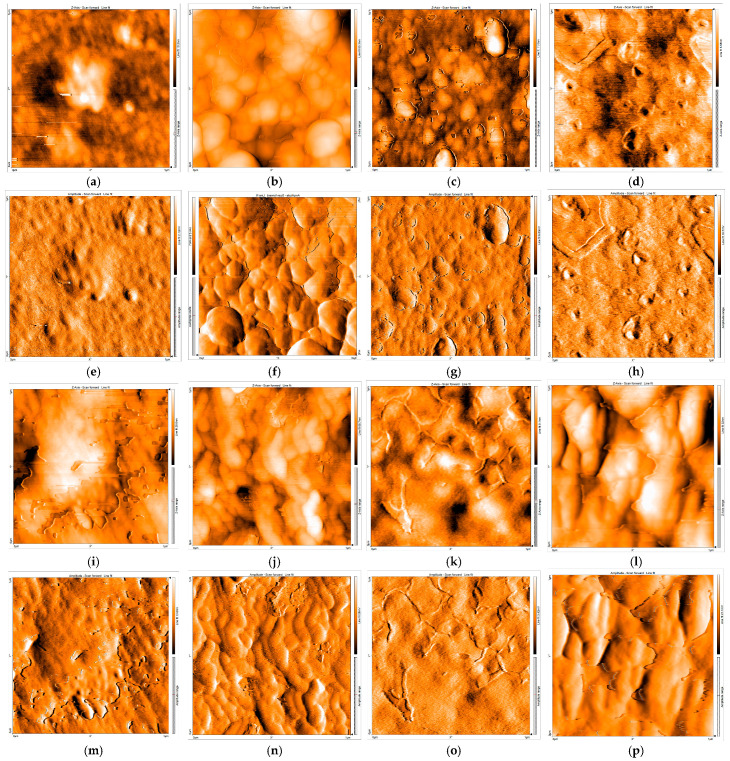
AFM images of free surfaces of the CoPUIALT900 films prepared under different conditions: heating at 170 °C (**a**,**e**); thermolysis at 350 °C (**b**,**f**); heating at 170 °C, 30 min, and hydrolysis in 0.04 N KOH, 2 days (**c**,**g**); thermolysis at 350 °C, 5 min, and hydrolysis in 0.04 N KOH, 1 day (**d**,**h**); (**a**–**d**)—topography, and (**e**–**h**)—amplitude, as well as AFM images of free surfaces of the CoPUIALT900/2000PCL films prepared under different conditions: heating at 170 °C (**i**,**m**); thermolysis at 350 °C (**j**,**n**); heating at 170 °C, 30 min, and hydrolysis in 0.04 N KOH, 2 days (**k**,**o**); thermolysis at 350 °C, 5 min, and hydrolysis in 0.04 N KOH, 1 day (**l**,**p**); (**i**–**l**)—topography, and (**m**–**p**)—amplitude; matrix scanning—1 × 1 µm.

**Figure 3:**
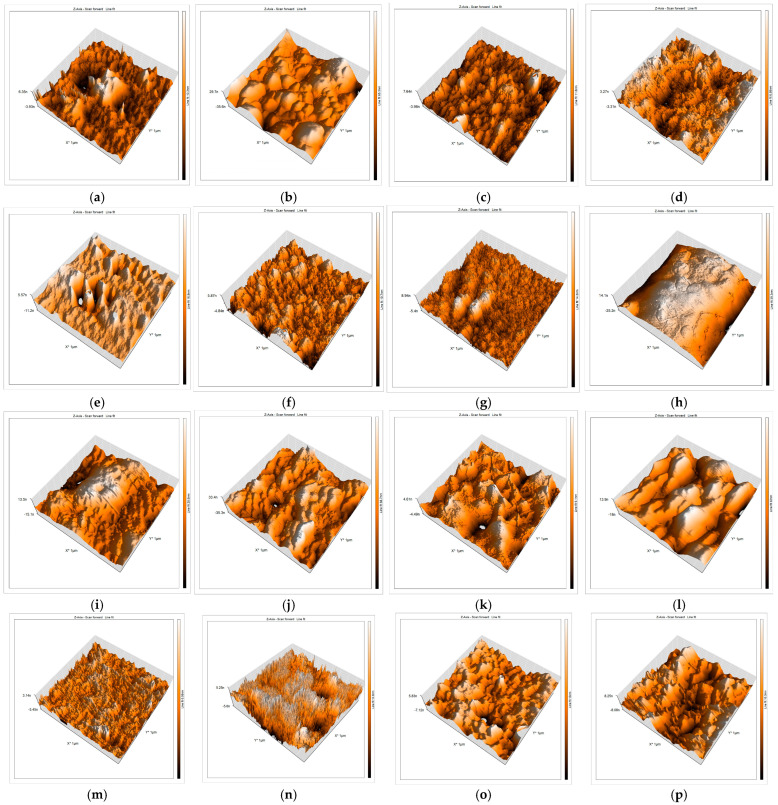
Three-dimensional images of free surfaces (**a**–**d**,**i**–**l**) as well as surfaces to the glass substrate (**e**–**h**, **m**–**p**) of the CoPUIALT900 films (**a**–**h**) and the CoPUIALT900/2000PCL films (**i**–**p**) prepared under different conditions: heating at 170 °C (**a**,**e**,**i**,**m**); thermolysis at 350 °C (**b**,**f**,**j**,**n**); heating at 170 °C, 30 min, and hydrolysis in 0.04 N KOH, 2 days (**c**,**g**,**k**,**o**); thermolysis at 350 °C, 5 min, and hydrolysis in 0.04 N KOH, 1 day (**d**,**h**,**l**,**p**); matrix scanning—1 × 1 µm.

When shooting higher-resolution AFM images of the fine structure of the upper surface of the CoPUIALT900 film, heated at 170 °C (Figure 2a,e), (a—topography, e—amplitude, scanning matrix 1 × 1 µm), a fine-grained morphology with grain sizes from 30 to 65 nm is clearly visible. Small gaps and pores with a diameter of less than 10 nm, which are localized in the near-surface layer of the sample, are more visible between the grains as shown in the 3D images (Figure 3a,e). The values of the arithmetic mean and root mean square roughness of the upper surface (for a 1 × 1 µm scan matrix) are Ra = 8.9 nm and Rq = 10.2 nm, respectively.

The lower surface of the CoPUIALT900 film, which was in contact with the glass substrate during preparation (Figure 3e), has a smoother relief compared to the upper surface; for a 1 × 1 µm matrix (Figure 3e), the roughness values are Ra = 1.4 nm and Rq = 1.8 nm. The grain boundaries are diffuse (Figure 2e), irregular scratches and defects (irregularities of irregular shape) are more visible in Figure S1(1B), and the roughness values are Ra = 27.3 nm and Rq = 31.9 nm for a 20 × 20 µm scanning matrix.

Subsequent thermolysis at 350 °C for 5 min of the copolymer CoPUIALT900 film radically changes the structure and morphology of the sample. At the same time, morphological changes are clearly visible on both surfaces of the CoPUIALT900 film (Figure S1(2A,B) and Figure 2b,f). The AFM images displayed rounded formations with a shape close to spherical on both surfaces of the thermolized sample—domains range in size from 80 to 300 nm (upper surface) and from 40 to 100 nm (lower surface). The domain morphology is most pronounced on the upper surface of the CoPUIALT900 sample, heated at 350 °C for 5 min (Figure 2b,f and Figure 3b). These domains may be the result of the release of gaseous decomposition products of degraded urethane fragments to the surface.

On the lower surface of the sample (to the glass substrate), the domain sizes are smaller than on the upper one (Figure 3f), while on both surfaces, inter-domain gaps in the form of slits with a width of less than 10 nm are detected between the domains.

It should be noted that the free surface of this sample, when the scanning matrix is 20 × 20 µm, has a highly developed relief. Nano-, meso- and micropores with sizes from 100–300 nm to 1–5 µm are evenly distributed over the entire surface (Figure S1(2A)). It is also possible to see spherical particles having a bright light contrast in the AFM image, measuring 300–400 nm, combined into aggregates of 10–20 particles. Such a relief may be caused by the “foaming” of the near-surface layers of the sample because of the diffusion of gaseous decomposition products of urethane fragments to the surface of the sample. Then, the non-burst bubbles form a “quasi-domain” morphology of the film surface and the burst ones form large pores in the near-surface areas of the sample and smaller pores in the volume.

In order to study the effect of hydrolytic processes on the morphology of the synthesized membrane copolymers, CoPUI samples heated at 170 °C and thermolized at 350 °C were subjected to alkaline hydrolysis. For this purpose, several compositions of a hydrolysis mixture of different concentrations were prepared and copolymer films (urethane imides) were treated for 1, 2 and 3 days. The etching technique is described in detail in our previous work [15].

It is interesting that after alkaline hydrolysis at 0.04 N KOH for 2 days, the relief of both surfaces of the copolymer CoPUIALT900 film, preheated at 170 °C, changed significantly. Clearly distinguishable oval domains with a diameter of 50 to 150 nm with clearly defined contours in the form of slits around each domain were observed (Figure 2c,g and Figure 3c,g). In addition, AFM images for the 20 × 20 µm scanning matrix (Figure S1(3A,B)) show a large number of spherical particles with dimensions of 100–300 nm. At the same time, the surface roughness decreased by more than 30–40 times for the upper surface, and by 10–15 times for the lower surface. Such a sharp smoothing of the relief of both CoPUIALT900 film surfaces is apparently caused not only by the etching effect of the surface layers of the film, but also by the process of transformation of the structure, resembling the effect of thermolysis at 350 °C of this sample.

The alkaline hydrolysis of a copolymer sample preliminarily thermolized at 350 °C leads to more significant morphological changes: dense inclusions of micron sizes are visible on the upper surface of the CoPUIALT900 film, which are apparently aggregates of crystallites (Figure S1(4A)). At a higher resolution (1 × 1 µm scanning matrix) (Figure 2d,h), large plate-like, apparently crystalline formations, can be seen on the upper surface of the sample—in the upper part of Figure 2d,h, two isolated crystalline aggregates grown via the mechanism of screw dislocations can be seen, as evidenced by the terraced morphology of these aggregates. Crystalline formations of lamellar morphology with angles between adjacent sides in crystals of 60 or 120° were also found on the lower surface (Figure 3h), which gives grounds to attribute these formations to the crystalline phase symmetry.

Let us proceed to the analysis of the morphology of copolymer films of a more complex composition, i.e., CoPUIALT900/2000PCL, containing aliphatic junctions in two types of macromolecules—ALT900 (M = 900 Da) and PCL2000 (M = 2000 Da).

AFM images of fragments of the upper (free) and lower (to the glass substrate) surfaces of the copolymer CoPUIALT900/2000PCL films (1 × 1 µm scanning matrix), at different stages of heat treatment (at 170 °C and 350 °C), are shown in Figure 2i,m,j,n. It can be recognized that all the domains have a non-uniform thickness, no regular porosity has been detected for these films, and individual defects in the form of cavities and slit gaps are observed, which are more clearly detectable on the upper film surfaces. When analyzing the AFM images obtained during scanning at a 20 × 20 µm matrix, it is also clear that the upper (free) and lower (to the glass substrate) surfaces of the CoPUIALT900/2000PCL films are micro heterogenic (Figure S1(5A,B and 6A,B and 7A,B and 8A,B)).

The domain morphology is visible for the CoPUIALT900/2000PCL films heated at 170 °C, when shooting high-resolution AFM images of a free surface (scanning matrix—1 × 1 µm) (Figure 2i,m). All the domains have an elongated elliptical shape with the following dimensions—width from 50 to 80 nm, with a length of about 300–500 nm (Figure 2i,m). The relief is comparatively rough, reaching roughness values of Ra = 4.94 nm and Rq = 5.84 nm. As shown by the analysis of the profile of the selected surface area, the domains rise above the level of the “average surface” by an average of 8–10 nm, and the height difference between the domain aggregates and the film surface can be 20–30 nm, which leads to the formation of a rough relief of this sample surface.

The CoPUIALT900/2000PCL film side to the substrate surface is very smooth (Figure 3m), the boundaries between the grains are practically invisible and individual inclusions have dimensions of the order of 50 nm. The roughness parameters for the 1 × 1 µm scanning matrix are Ra = 1.20 nm and Rq = 1.36 nm.

Thermolysis at 350 °C of the CoPUIALT900/2000PCL sample leads to a noticeable transformation of the morphology of this film. The upper surface has an unusual pronounced domain morphology with curved elongated elliptical domains, rather tightly adjacent to each other (Figure 2j,n), 50–100 nm wide and 200–400 nm long. The roughness parameters for the 1 × 1 µm scanning matrix are Ra = 8.67 nm and Rq = 10.43 nm. The height difference of the domains above the film plane is 30 nm (Figure 3j). At the same time, the lower surface of this film is smoother, and separate isolated large pores of the order of 100 nm are visible on it. The roughness is Ra = 4.91 nm and Rq = 5.61 nm (Figure 3n).

It is interesting that after alkaline hydrolysis in 0.04 N KOH of the CoPUIALT900/2000PCL films preheated at 170 °C, structural changes occur, leading to the formation of a much smoother surface, which is formed by flattened elongated domains with dimensions ranging from 150 nm wide, up to 400 nm long (Figure 2k,o), and the surface roughness is Ra = 8.57 nm and Rq = 9.80 nm.

A fine-grained morphology with grain diameters of 30–50 nm was found on the lower surface of the CoPUIALT900/2000PCL film. The grains are combined into chains up to 200 nm long; the relief height is 3–5 nm. The surface of the film is very smooth, where the roughness is Ra = 1.87 nm and Rq = 2.14 nm (Figure 2k,o).

Hydrolysis in a solution of 0.04 N KOH for 2 days of a CoPUIALT900/2000PCL sample thermolized at 350 °C leads to noticeable changes in the morphology of the upper surface (Figure 2l,p) with very large elliptical domains 50–150 nm wide, 300–700 nm long, with slit-like gaps between them. The elevation difference is 25 nm, while the roughness is Ra = 3.81 nm and Rq = 4.31 nm. Grains of 30–80 nm in size are visible on the lower surface; the elevation difference is 15 nm, while the roughness is Ra = 4.80 nm and Rq = 6.10 nm (Figure 3p). Apparently, during the hydrolysis of the thermolized sample, the processes of separation of the products of destruction of the copolymer macro chains and their diffusion to the surface of the film also occur, which leads to swelling of the domain formations and a certain increase in their sizes.

Thus, it has been shown that after alkaline hydrolysis, the roughness values of both surfaces of films heated at 170 °C decrease significantly—almost 30–40 times for the upper surface and 10–15 times for the lower surface. Such a sharp smoothing of the relief of both film surfaces after hydrolysis is caused not only by the etching effect of the surface layers of the film, but also by the process of transformation of the structure. It is shown that during the hydrolysis of the thermolized samples at temperatures of 300 °C or 350 °C, the processes of separation of the products of destruction of urethane fragments and their diffusion to the surface of the films also occur, which leads to the “swelling” of domain formations and a certain increase in their size.

### 3.5. Scanning Electron Microscopy (SEM) Investigations of the CoPUI Films

Scanning electron microscopy (SEM) was used to determine the distribution of nano- and microporosity on the surface of the films of synthesized copoly(urethane-imides). The free surfaces of films and the surfaces of the low-temperature sections of the samples obtained at the temperature of liquid nitrogen were studied.

It should be noted that copolymer films, even those subjected to high-temperature heating—thermolysis—have an amorphous phase in their composition, which is localized in the gaps between ordered domains or crystalline formations, and also covers the surface of samples, leading to a smoothing of the relief and blurring of the contours of supramolecular formations.

To enhance the contrast in SEM research, selective etching techniques are usually used, when an amorphous layer is removed from the surface of the polymer film, leaving the crystalline regions untouched. In this case, a high contrast occurs, which allows the visualization of the elements of the supramolecular structure of the films and determination of their sizes.

Previously, we have usually used the technique of selective etching of polymer materials using solutions of potassium permanganate in an acidic medium (orthophosphoric or sulfuric acid [29]. In this work, we also applied the technique of etching the surface of films with a solution of potassium permanganate in orthophosphoric acid to remove a thin surface layer from the samples and visualize the porous structure.

**Figure 4:**
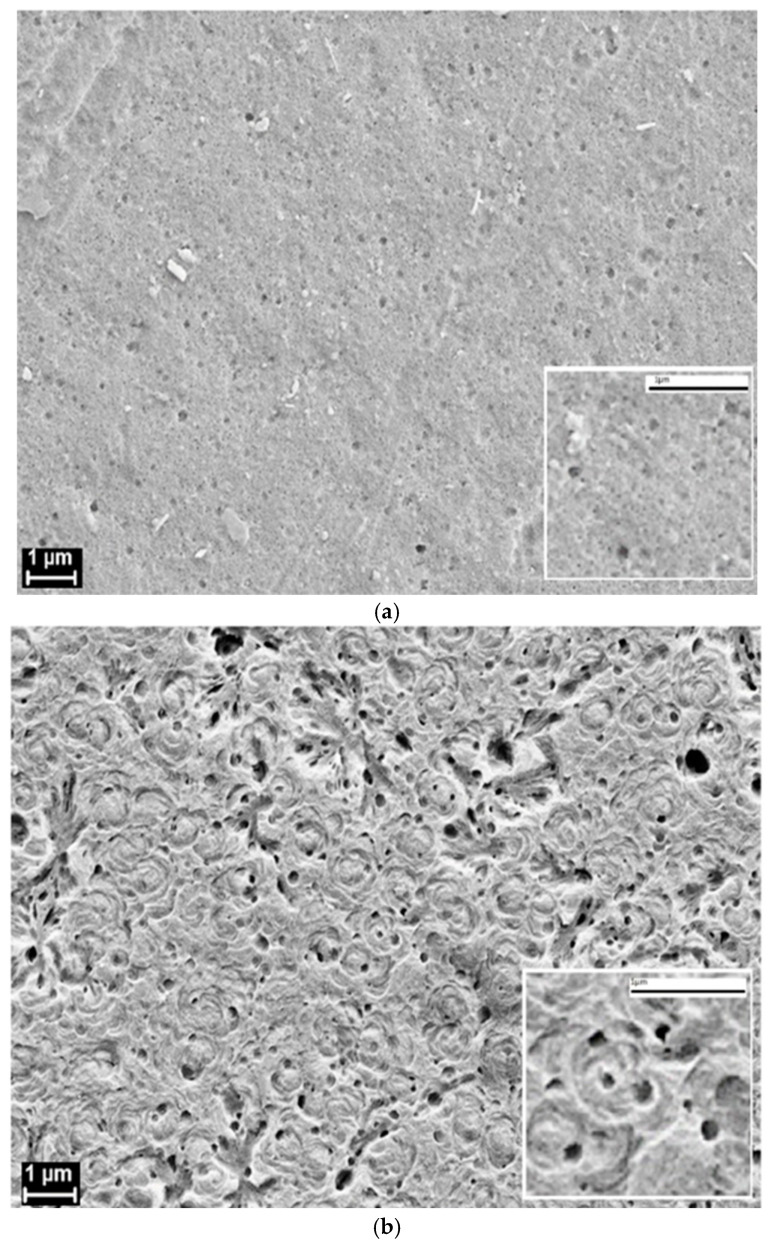
SEM micrographs of the surface of copolymer films after thermolysis at 350 °C and hydrolysis in 0.04 N KOH, etched with a solution of potassium permanganate: (**a**)—CoPUIALT900, (**b**)—CoPUIALT900/2000PCL. The insert shows an enlarged image fragment.

Figure 4 shows scanning electron micrographs of the surface of the CoPUIALT900 film after thermolysis at 350 °C and hydrolysis in 0.04 N KOH (a) and CoPUIALT900/2000PCL film, and also after thermolysis at 350 °C and hydrolysis in 0.04 N KOH (b). It can be seen that the CoPUIALT900 sample exhibits nanoporous morphology. On its etched surface, there is a large number of small pores ranging in size from 5 to 20 nm, almost uniformly distributed over the entire film surface at distances of about 40–50 nm from each other. The analysis of the micrograph in Figure 4a allows the conclusion that the pore size distribution is a quite narrow unimodal distribution. In addition, separate crater depressions with a diameter of about 200 nm are observed, and on the surface of the sample at distances of 400–600 nm between them, there are apparently traces of etched gas bubbles (decomposition products of urethane fragments) that have separated from the volume of the film and diffused to the surface of the sample.

These data are in good agreement with the results obtained by using the AFM method, according to which a fairly uniform porosity was found on the free surface of this sample (for a 20 × 20 µm scanning matrix), and uniformly distributed nano-, meso- and micropores with sizes from 100–300 nm to 1–5 µm are observed on it (Figure S1(4A,B)).

The CoPUIALT900/2000PCL sample (Figure 4b) radically differs in morphology from the previous sample. The surface of the sample is highly textured, and a large number of spherical formations are observed on it, consisting of lamellae wrapped around the center, measuring from 400 to 1000 nm, shaped like a cabbage. In the center of almost every such sphere (lamellar “head”) there is a pore measuring about 100–200 nm. An analysis of the general nature of the porosity of this sample showed that the pore size distribution is much wider than in the previous sample, the pores are larger, and in addition, interlamellar gaps and cracks formed because of lamella delamination contributing to the overall porosity.

SEM micrographs of the fracture surface of the CoPUIALT900 copolymer film, subjected to thermolysis at 350 °C and hydrolysis in 0.04 N KOH are shown in Figure S2a,b of the Supplementary Materials. Here, all the film volume exhibits many nanopores with sizes of 10–20 nm (Figure S2b). Larger micropores of about 200–400 nm in size are also occasionally found.

Thus, the SEM method has shown that the synthesized films of CoPUIs after thermolysis and hydrolysis in KOH solution are porous materials with significantly different sizes and characters of pore size distribution as well as supramolecular structures of the films. The size range of pores varies from several nanometers to hundreds of nanometers in the films under study, which makes it possible to use these CoPUIs as membranes for nano- and microfiltration.

### 3.6. X-Ray Diffraction (XRD) Investigation of the CoPUI Films

The prepared CoPUI films after imidization at 170 °C, subsequent thermolysis and hydrolysis were examined via X-ray diffractometry (XRD).

**Figure 5:**
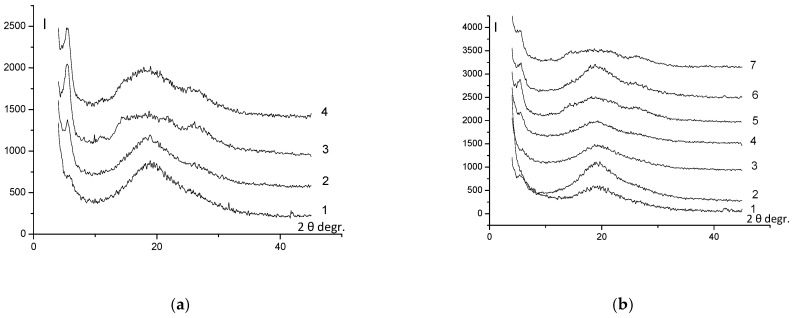
(**a**) Diffractograms from CoPUIALT900 films with different processing techniques: 1—heating at 170 °C; 2—thermolysis at 300°С, 30 min; 3—thermolysis at 350 °C, 5 min and hydrolysis in 0.04 N KOH, 1 day; 4—thermolysis at 350 °C, 5 min; (**b**) diffractograms from CoPUIALT900/2000PCL with different processing techniques: 1—heating at 170 °C; 2—heating at 170 °C, 30 min and hydrolysis in 0.04 N KOH, 1 day; 3—heating at 170 °C, 30 min and hydrolysis in 0.04 N KOH, 2 days; 4—thermolysis at 300 °C, 30 min and hydrolysis in 0.04 N KOH, 2 days; 5—thermolysis at 350 °C, 5 min and hydrolysis in 0.04 N KOH, 1 day; 6—thermolysis at 300 °C, 30 min; 7—thermolysis at 350 °C, 5 min.

The diffractogram of the CoPUIALT900 film, heated at 170 °C, shows one diffuse reflection (amorphous halo) at 2*θ*~19° and one very weak reflection at 2*θ*~5.42°, which indicate its practically amorphous structure (Figure 5a, curve 1). The thermolysis of the copolymer film at 300 °C (curve 2 in Figure 5a) leads to the considerable increasing of the intensity of the reflection at 2*θ*~5.42° (approx. two-fold), and the appearance of one additional comparatively weak reflection at 2*θ*~26.1°, which form “shoulders” against the main amorphous halo. This indicates that the processes of ordering and formation of crystalline phase nuclei of small sizes take place in the film.

After the thermolysis of the CoPUIALT900 sample at 350 °C, 5 min (curve 4 in Figure 5a), a drastically increasing reflection intensity at 2*θ*~5.42° (approx. five-fold) is observed. Moreover, three diffuse reflections at 2*θ*~10.82°, 14.29° and 21.78° and a weak reflection at 2*θ*~26.1° can be recognized in the amorphous halo background which evidences the perfection of the crystalline structure of this film and increase in the crystallites sizes.

Subsequent hydrolysis in 0.04 N KOH during 1 day of preheating at 170 °C of the CoPUIALT900 sample does not change its structure. The copolymer film remains amorphous, because only an amorphous halo can be seen in its diffractogram.

On the other hand, the subsequent hydrolysis of the CoPUIALT900 film (preliminarily thermolized at 350 °C) in 0.04 N KOH (curve 3 in Figure 5a) leads to a more ordered structure, because diffusion reflections at 2*θ*~10.82°, 14.29° and 21.78°, and 26.1° became more pronounced on the diffractogram.

The diffractogram from the CoPUIALT900/2000PCL films heated at 170 °C shows one diffuse reflection (amorphous halo) at 2*θ*~19°, and the same very weak reflection at 2*θ*~5.5° (Figure 5b, curve 1), as in the previous copolymer sample heated at 170 °C. This reflection disappeared after hydrolysis in 0.04 N KOH during 1 day of preheating at 170 °C of the copolymer CoPUIALT900/2000PCL films (Figure 5b, curve 2) or 2 days (Figure 5b, curve 3) which means that the sample still exists in an amorphous state.

The thermolysis of the copolymer film at 300 °C for 30 min (curve 6 in Figure 5b) and 350 °C for 5 min (curve 7 in Figure 5b) leads to a slight increase in the intensity of the reflections at 2*θ*~5.5°, 14.5°, 22° and ~26°, which evidences the formation of the crystalline structure in the sample, but with very small crystallite sizes.

Hydrolysis in 0.04 N KOH for 2 days of CoPUIALT900/2000PCL films preliminarily thermolized at 300 °C (curve 4 in Figure 5b) and 350 °C in 0.04 N KOH (curve 5 in Figure 5b) leads to a less ordered structure—only very small diffusion reflections can be detected in the diffractogram of the hydrolyzed CoPUIALT900/2000PCL sample.

A comparison of the angular positions of reflections at 2*θ*~5.42°, 10.82°, 14.29°, 21.78° and 26.1° with angular positions of reflections from thermally imidized PMDA-ODA powder of 2*θ*~14.9, 22.2, 26.5° [12] shows that the formation and growth of aromatic-phase crystallites occur during thermolysis at 300–350 °C. There are no reflections from crystallites of the aliphatic phase—polycaprolactone or alt/polycaprolactone have been detected in the diffractograms of hydrolyzed CoPUIALT900 and CoPUIALT900/2000PCL samples. It should be noted that the main very intense and narrow crystalline reflections of polycaprolactone at 2*θ*~21.3 and 23.5° located in the area close to the angle position of the amorphous halo at 2*θ* = 20° were not revealed in the diffractograms of the thermolized samples.

XRD studies of copoly(urethane-imides) with different processing techniques at different stages of chemical transformations confirm the course of selective destruction of aliphatic (urethane) blocks and their subsequent removal from copolymer films during thermolysis in the air and hydrolysis in alkali media, which coincides well with our results obtained in previous works [15,16].

### 3.7. DSC, TGA, DMA and Deformation—Strength Investigation

In the work, the polymers were tested in the form of film samples, and the initial films (cured at 170 °C) had high strength and elasticity (Table 2).

It is appropriate to note that in their chemical structure, the aliphatic polyols used in the preparation of copolymers, in their chemical structure, are polyesters of oxyacid (PCL2000) and polyesters formed by diol and dicarboxylic acid (ALT900), terminated by hydroxyl groups, respectively, and differ sharply in their molecular weight (2000 vs. 900). It can be seen that the transition from PLC to ALT in the composition of the copolymer, as well as a decrease in the length of the aliphatic fragment in it, reduces the elongation and elasticity of the copolymer, while increasing the strength of the film sample.

Since the target products in the work are obtained as a result of the use of destructive processes (selective thermolysis and alkaline hydrolysis), it is advisable to give the properties of films after the thermolysis operation and show how the properties change as a result of the subsequent alkaline hydrolysis (Table 3 and Table 4).

From the comparison of the data in Table 2 and Table 3, it follows that because of the thermolysis process, the elongation values at the break of the samples decrease, while the values of Young’s modulus, yield and tensile strength increase. This is especially noticeable in the case of the CoPUIALT900 sample, which was thermolized at 350 °C. In this case, the values of the deformation and strength parameters approach the values for basic poly(4,4′-oxydiphenylene)pyromellitimide films.

As follows from the data in Table 4, the tightening of the alkaline hydrolysis conditions of thermolized samples (temperature and exposure time in baths) leads to a significant increase in the deformation and strength properties of the film samples: reinforcement of the strength and weakening of the elastic properties. The observed effect is probably related to the removal of flexible aliphatic links from the copolymers, which very likely play the role of plasticizers. The maximum effect is observed in the case of the CoPUIALT900 sample, thermolized at 350 °C and etched in an alkaline solution for 12 h: E = 4.01 GPa and ε = 5.7%. It should be noted that the indicators of deformation and strength properties of the targeted polymers presented in Table 4 are equivalent to the corresponding characteristics of classical heat-resistant polyimides.

The thermal stability and heat resistance of the synthesized samples were evaluated using the methods of TGA, DSC and DMA. The values of the thermal stability index τ_5_ (temperature corresponding to a 5% weight loss of the sample during TGA) for a set of initial films cured at 170 °C lie in the range of 309–394 °C, films thermolized at 300 °C lie in the range of 410–421 °C, the target films hydrolyzed after thermolysis at 300 °C vary within the range of 364–425 °C and the target films hydrolyzed after 350 °C thermolysis lie in the range of 376–445 °C. The observed changes indicate the enrichment of the polymer systems under consideration with aromatic fragments.

The noted pattern is manifested in the study of polymers by using the DSC method. From the analysis of the low-temperature regions of the DSC curves of the first and second scans, it follows that the aliphatic blocks of the initial copolymers are characterized by a glass transition temperature T_g_ = 6.2 °C and a melting point T_m_ = 65 °C in the case of copolymer CoPUI2000PCL, T_g_ = 5 °C and T_m_= 70 °C in the case of copolymer CoPUIALT900/2000PCL, and T_g_ = 3.8 °C and T_m_= 58.6 °C in the case of copolymer CoPUIALT900. Usually, in all cases of alkaline hydrolysis, the glass transition and melting point of the aliphatic phase of the samples were not detected. The results of the study are summarized in Table S2 (in the Supplementary Materials).

**Figure 6:**
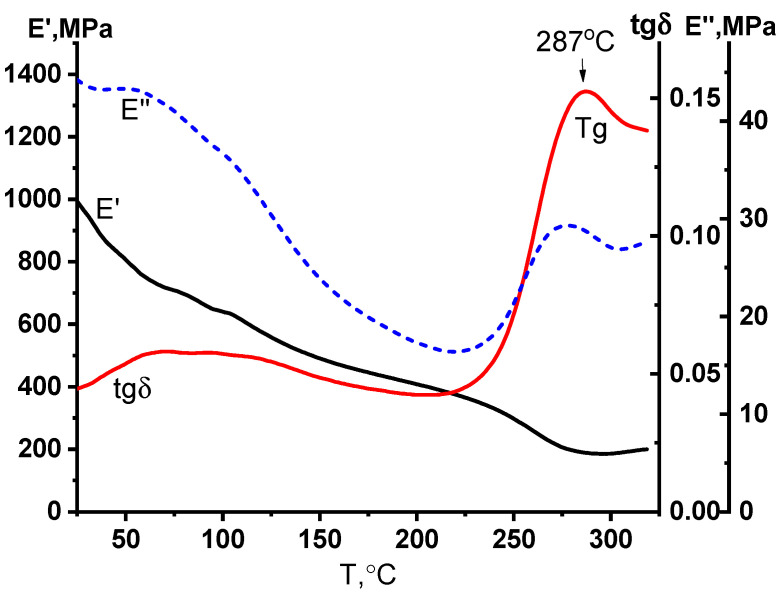
DMA curves of copoly(urethane-imide) samples CoPUIALT900/2000PCL thermolized for 30 min at a temperature of 300 °C. Temperature dependencies E′ (red curve), E″ (black curve) and tan δ (blue curve).

Figure 6 shows an example the DMA curves for the CoPUIALT900/2000PCL sample thermolized at 300 °C for 30 min. The glass transition temperature of aromatic blocks in accordance with the maximum of the temperature dependence of the loss tangent is T_g_ = 287 °C. There is an inflection in the low-temperature region of the curve, which can be explained by the presence of thermal degradation products of aliphatic blocks in the sample that has not been hydrolyzed. According to the DMA data, the target polymers (after alkaline hydrolysis) are characterized by high T_g_ values lying in the range of 265–401 °C.

So, the removal of polyurethane links from the composition of macromolecules of copoly(urethane-imide)s is also indirectly evidenced by the differences in heat resistance, as well as mechanical properties determined in static and dynamic test modes.

## 4. Conclusions

In the present work, the structure, morphology, heat resistance and mechanical properties of polyimide porous films prepared as a result of successive processes of thermolysis and hydrolysis in alkaline solutions of the initial copolymer films (urethane-imides) of the new composition are investigated. The initial copolymers contained rigid blocks of poly(4,4′-oxidiphenylene)pyromellitimide and soft blocks of segmented copoly(urethane-imides) based on polycaprolactone (PCL, Mn = 2000) and poly(1,6 hexanediol/neopentylglycol-alt-adipic acid) (ALT, Mn = 900), respectively. The molar ratio of hard and soft blocks was 10:1. The prepared porous polyimide films had a complex specific morphology, depending both on the composition of the initial copolymer and on the conditions of subsequent thermal and chemical treatment—thermolysis at a temperature of 300 °C or 350 °C, and then hydrolysis in a bath containing an aqueous solution of potassium hydroxide.

The structural and morphological characteristics of the synthesized copolymers films have been studied via AFM, SEM and X-ray diffraction. Significant differences were found in the morphology of the surface of films containing links of one or both polymer diols in macromolecules. It is shown that the roughness of the upper (free) surfaces of the prepared CoPUI films formed during formation from the solution is several times higher than the roughness of the surface, which was in contact with the glass substrate during the process of production of the films.

It was found that the transition from PLC to ALT aliphatic fragments in the CoPUIs, as well as a decrease in their length, increases the strength of the CoPUI film samples, while reducing the elongation and elasticity of the copolymer. At the same time, the thermolysis process increased the values of Young’s modulus, yield strength and tensile strength, but the elongation at break decreased. This is especially noticeable in the case of the CoPUIALT900 sample, which was thermolized at 350 °C. The thermolysis temperature rise and increased exposure time in baths of the alkaline hydrolysis led to a significant rise in the deformation and strength properties of the film samples and declines in the elastic properties of films. The maximum effect was also observed in the case of the CoPUIALT900 sample, thermolized at 350 °C and etched in an alkaline solution for 12 h.

The results obtained show that the developed technique of selective destruction of soft urethane blocks of copoly(urethane-imide)s via thermolysis and subsequent hydrolysis in alkaline media is effective for obtaining new porous polymer systems for the manufacture of micro- and nanofiltration membranes for high-temperature applications.

## Figures and Tables

**Table 1:** Designations of the synthesized copolymers.

CoPUI Samples	Abbreviations	The Molar Ratio of the Starting Substances				
		PM	TDI	PCL2000	ALT900	ODA
(PM-TDI-PCL2000-TDI-PM)(PM-ODA)_10_	CoPUI2000PCL	12	2	1	0	10
(PM-TDI-(ALT900)_0.5_-(PCL2000)_0.5_-TDI)(PM-ODA)_10_	CoPUIALT900/2000PCL	12	2	0.5	0.5	10
(PM-TDI-ALT900-TDI-PM)(PM-ODA)_10_	CoPUIALT900	12	2	0	1	10

**Table 2:** Deformation and strength properties of films of synthesized copoly(urethane-imide)s before the thermolysis (final curing temperature is 170 °C).

Sample	E, GPa	σ, MPa	ε, %
CoPUI2000PCL, 170 °C	1.76 ± 0.05	33 ± 5	172 ± 8
CoPUIALT900/2000PCL, 170 °C	2.51 ± 0.02	97 ± 3	153 ± 4
CoPUIALT900, 170 °C	2.80 ± 0.01	118 ± 8	115 ± 12

**Table 3:** Comparison of deformation and strength properties of films of copoly(urethane-imide)s with different compositions of aliphatic units as a result of thermolysis (before alkaline hydrolysis).

Temperature and Duration of Thermolysis	E, GPa	σ, MPa	ε, %
**CoPUI2000PCL**			
300 °C, 30 min	1.82 ± 0.07	44 ± 7	147 ± 4
350 °C, 5 min	1.97 ± 0.01	56 ± 4	139 ± 3
**CoPUIALT900/2000PCL**			
300 °C, 30 min	2.50 ± 0.05	99 ± 4	52 ± 1
350 °C, 5 min	2.88 ± 0.09	129 ± 10	79 ± 2
**CoPUIALT900**			
300 °C, 30 min	2.99 ± 0.06	123 ± 2	52 ± 1
350 °C, 5 min	3.22 ± 0.12	123 ± 5	34 ± 2

**Table 4:** Deformation and strength properties of the samples subjected to hydrolysis in aqueous 0.04 N solutions of potassium hydroxide at a temperature of 20 °C with copolymer films preliminarily thermolized at temperatures of 170, 300 and 350 °C.

The Thermolysis Temperature and the Holding Time of the Samples in an Alkali Solution	E, GPa	σ, MPa	ε, %
**CoPUI2000PCL**			
170 °C, 24 h	2.68 ± 0.29	123 ± 15	140 ± 15
170 °C, 48 h	2.47 ± 0.23	73 ± 8	18 ± 1
300 °C, 24 h	2.02 ± 0.07	107 ± 10	96 ± 8
300 °C, 48 h	2.10 ± 0.01	113 ± 4	112 ± 11
300 °C, 72 h	2.00 ± 0.04	100 ± 2	92 ± 2
350 °C, 24 h	2.50 ± 0.08	118 ± 4	74 ± 8
350 °C, 48 h	2.27 ± 0.07	105 ± 7	73 ± 15
**CoPUIALT900/2000PCL**			
170 °C, 24 h	2.68 ± 0.29	123 ± 12	140 ± 13
170 °C, 48 h	2.47 ± 0.23	73 ± 8	18 ± 2
300 °C, 12 h	2.55 ± 0.17	83 ± 2	7 ± 1
350 °C, 6 h	2.71 ± 0.09	76 ± 1	5 ± 1
**CoPUIALT900**			
170 °C, 6 h	1.66 ± 0.32	57 ± 1	6 ± 1
300 °C, 24 h	3.11 ± 0.17	84 ± 4	9 ± 1
300 °C, 48 h	3.21 ± 0.04	109 ± 11	21 ± 1
300 °C, 72 h	3.06 ± 0.27	107 ± 9	29 ± 2
350 °C, 12 h	4.01 ± 0.11	111 ± 1	6 ± 1

## Data Availability

The source data presented in the study is publicly available. The data that support the findings of this study are available from the corresponding author upon reasonable request.

## Supplementary Materials

The following supporting information can be downloaded at: https://www.mdpi.com/article/10.3390/polym17030329/s1, Table S1: The combined table of the NMR study; Figure S1: AFM images of the free surface and the surface to the glass substrate of CoPUIALT900 and (CoPUIALT900/2000PCL) films after heat treatment at 170 °C and after thermolysis at 350 °C, as well as after thermolysis and subsequent alkaline hydrolysis; amplitude mode; scanning matrix 20 × 20 µm; Table S2: TGA and DSC results of the study of film samples: CoPUI2000PCL, CoPUIALT900/2000PCL and CoPUIALT900; Figure S2: SEM images of the fracture surface CoPUIALT900 film after thermolysis at 350 °C and subsequent alkaline hydrolysis; magnification: ×1000 (a), ×10.000 (b).
