# Supplementary material for: Structure–Properties Correlations in Novel Copoly(urethane-imide) Films Selectively Destructed Under Thermolysis and Hydrolysis in Alkaline Media
Source: Polymers (Basel). 2025 Jan 25;17(3):329. doi: 10.3390/polym17030329 (PMC11820994; doi:10.3390/polym17030329)
Supplement: Supplementary file 1 [file polymers-17-00329-s001.zip › polymers-3383839-supplementary.pdf]

polymers

MDPI

Table S1. The combined table of the NMR study.

Structure	NMR, ppm
	CoPUI2000PCL
Prepolymer (*DMSO-d*[6])	9.96; 8.08; 7.80; 7.60; 7.51; 7.43; 7.30; 7. 17; 7.09; 7.02; 6.80; 4.17; 3.92; 3.60; 2.30; 2.24; 2.18; 1.64; 1.59; 1.30
CoPUI (*solid state*)	1.25; 1.63; 2.21; 4.03; 7.20; 8.29; 9.90
After thermolysis (300°C) (*solid state*)	1.27; 1.74; 2.19; 3.08; 5.34; 6.17; 7.11; 8.26
After alkaline hydrolysis (*solid state*)	1.21; 2.26; 3.13; 5.31; 6.20; 7.20; 8.28
	CoPUIALT900
Prepolymer (*DMSO-d*[6])	10.1; 8.05; 7.76; 7.54; 7.49; 7.36; 7.29; 7. 15; 7.04; 7.00; 6.84; 4.22; 3.98; 3.57; 2.41; 2.29; 1.93; 1.73; 1.67; 1.63; 1.46; 0.88
CoPUI (*solid state*)	0.89; 1.72; 2.17; 3.92; 7.17; 8.32; 9.96
After thermolysis (300°C) (*solid state*)	0.93; 1.27; 1.68; 2.10; 3.12; 5.26; 6.20; 7.12; 8.35
After alkaline hydrolysis (*solid state*)	1.20; 2.12; 3.10; 5.22; 6.34; 7.21; 8.28
	CoPUIALT900/2000PCL
Prepolymer (*DMSO-d*[6])	9.92; 8.09; 7.84; 7.61; 7.53; 7.40; 7.22; 7. 18; 6.99; 6.90; 6.78; 4.16; 3.96; 3.61; 2.38; 2.30; 2.23; 2.15; 1.87; 1.75; 1.64; 1.56; 1.40; 0.85
CoPUI (*solid state*)	0.89; 1.20; 1.66; 2.20; 3.99; 7.26; 8.36; 9.89
After thermolysis (300°C) (*solid state*)	0.90; 1.20; 1.66; 2.20; 3.99; 5.30; 6.19; 7.26; 8.36
After alkaline hydrolysis (*solid state*)	1.15; 2.08; 3.16; 5.31; 6.25; 7.30; 8.22

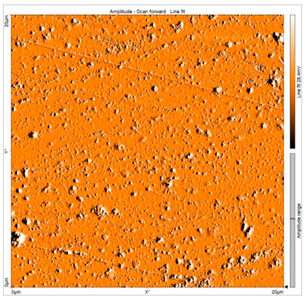

1A
CoPUIALT900, 170°C,
upp. surf. Ra = 168 nm
Rq = 195 nm

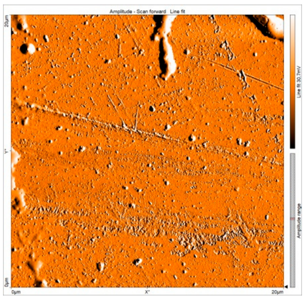

1B
CoPUIALT900, 170°C,
low. surf. Ra = 27.26 nm
Rq = 31.85 nm

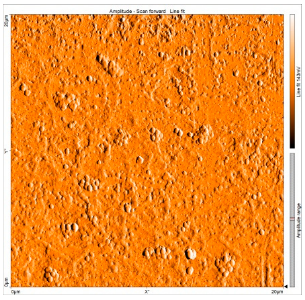

2A
CoPUIALT900, 350°C,
upp. surf. Ra = 248 nm
Rq = 317 nm

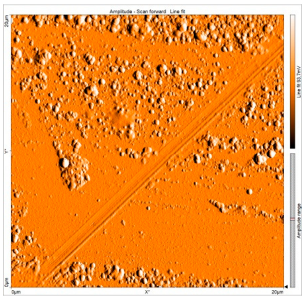

2B
CoPUIALT900, 350°C,
low. surf. Ra = 118 nm
Rq = 133 nm

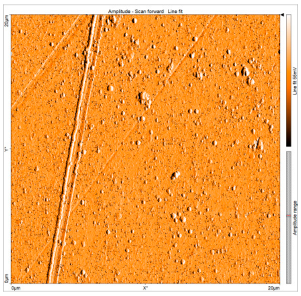

3A
CoPUIALT900, 170°C,
0.04 N KOH, upp. surf.

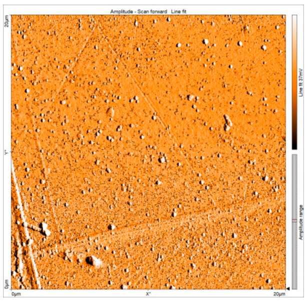

3B
CoPUIALT900, 170°C,
0.04 N KOH, low. surf.

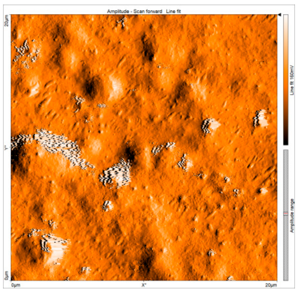

4A
CoPUIALT900, 350°C,
0.04 N KOH, upp. surf.

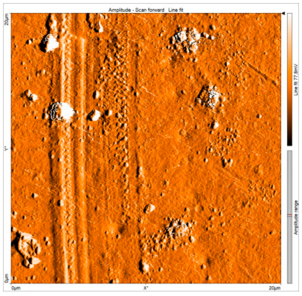

4B
CoPUIALT900, 350°C,
0.04 N KOH, low. surf.

Ra = 3.41 nm Rq = 6,26 nm	Ra = 1.38 nm Rq = 2,40 nm	Ra = 91.02 nm Rq = 125 nm	Ra = 11.62 nm Rq = 14.09 nm
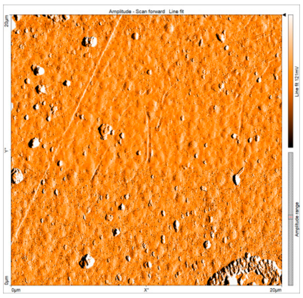	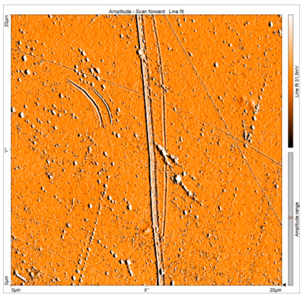	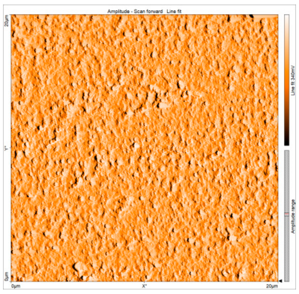	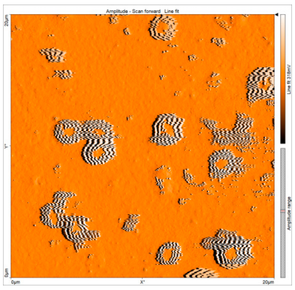
5A<br>***CoPUIALT900/2000PCL,***<br>170°C, upp. surf.<br>Ra = 21.22 nm Rq = 33.61 nm	5B<br>***CoPUIALT900/2000PCL,***<br>170°C low. surf.<br>Ra = 19.86 nm Rq = 22.74 nm	6A<br>***CoPUIALT900/2000PCL,***<br>350°C, upp. surf.<br>Ra = 181.5 nm Rq = 213 nm	6B<br>***CoPUIALT900/2000PCL,***<br>350°C, low. surf.<br>Ra = 53.49 nm Rq = 59.53 nm
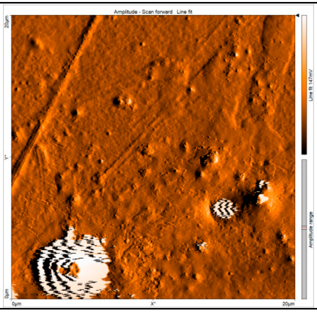	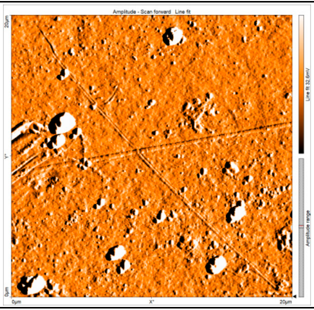	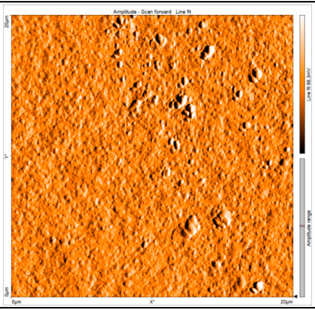	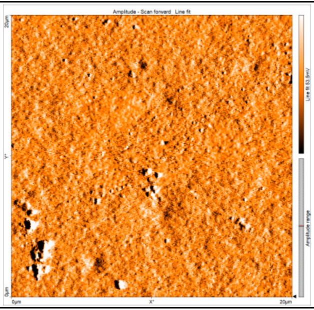
7A<br>***CoPUIALT900/2000PCL,***<br>170°C, 0.04 N KOH, upp. surf. Ra = 40.11 nm Rq = 47.53 nm	7B<br>***CoPUIALT900/2000PCL,***<br>170°C, 0.04 N KOH, low. surf. Ra = 176.8 nm Rq = 203.7 nm	8A<br>***CoPUIALT900/2000PCL,***<br>350°C, 0.04 N KOH, upp. surf. Ra = 71.84 nm Rq = 78.78 nm	8B<br>***CoPUIALT900/2000PCL,***<br>350°C, 0.04 N KOH, low. surf. Ra = 8.05 nm Rq = 9.62 nm

Figure S1. AFM images of the free surface and the surface to the glass substrate of ***CoPUIALT900*** and ***(CoPUIALT900/2000PCL)*** films after heat treatment at 170 °C and after thermolysis at 350 °C, as well as after thermolysis and subsequent alkaline hydrolysis; amplitude mode; scanning matrix 20 × 20 μm.

Table S2. TGA and DSC results of the study of film samples: *CoPUI2000PCL, CoPUI-ALT900/2000PCL and CoPUIALT900.*

The warm-up mode, the normality of the KOH solution and the etching time in it	Residual mass at 800°C,%	τ_5, °C	ΔH, J/g	T_m (1scan), °C	T_g (2scan), °C
		CoPUI2000PCL			
170 °C	40.12	353.7	7	65.0	6.2
170 °C, 0,5 N KOH, 24 h	43.9	342.0	10.1[1]; 8.1[2]	70[1]; 195[2]	5.1
170 °C, 1 N KOH, 6 ч	46.1	328.0	6.4[1]; 5.5[2]	83[1]; 199[2]	3.8
170 °C, 1 N KOH, 30 ч	46.1	324.0	5.9[1]; 5.7[2]	75[1]; 199[2]	0.1
170 °C, 2 N KOH, 6 h	45.8	327.0	8.6[1]; 5.8[2]	70[1]; 199[2]	4.8
170 °C, 2 N KOH, 12 h	46.1	326.0	7.2[1]; 6.1[2]	70[1]; 198[2]	3.7
300 °C, 2 N KOH,24 h	49.0	372.0	4,1	66.6	5.0
300 °C, 2 N KOH,48 h	48.7	364.0	-	-	-
350 °C, 2 N KOH, 24 h	48.3	376.0	-	-	-

350 °C, 2 N KOH, 48 h	49.8	376.0	-	-	-
		CoPUIALT900/2000PCL			
170 °C	46.13	393.6	7.8	70.0	5.0
300 °C	48.14	410.9	4.65	52.8	4.0
350 °C	51.65	422.2	10.21	69.6	4.5
170 °C, 0,04 N KOH, 24 h	47.49	349.0	7.8	70.0	5.0
170 °C, 0,04 N KOH, 48 h	46.35	351.0	4.8[1]; 4.45[2]	50.5[1];194,[2]	37.8[2]
300 ºC, 0,04 N KOH, 24 h	50.02	375.0	-	-	-
300 °C, 0,04 N KOH,72 h	48.84	366.0	-	-	-
350 °C, 0,04 N KOH, 24 h	53.8	395.0	-	-	-
350 °C, 0,04 N KOH,72 h	53.43	386.0	-	-	-
		CoPUIALT900			
170 °C	47.41	333.8	15.11	58.6	3.8
300 °C	52.64	421.3	9.1	53.3	4.3
350 °C	56.27	482.5	15.09	59.3	4.5
170 °C, 0,04 N KOH, 24 h	51.8	409.3	9.6[1]; 7.8[2]	47[1]; 189[2]	11.8
170 °C, 0,04 N KOH, 48 h	52.5	411.2	8.7[1]; 5.3[2]	49.3[1]; 191[2]	9.6
300 °C, 0,04 N KOH, 24 h	55.93	421.7	-	-	-
300 °C, 0,04 N KOH, 48 h	56.42	424.8	-	-	-
350 °C, 0,04 N KOH, 24 h	61.2	431.6	-	-	-
350 °C, 0,04 N KOH, 72 h	6.9	444.5	-	-	-

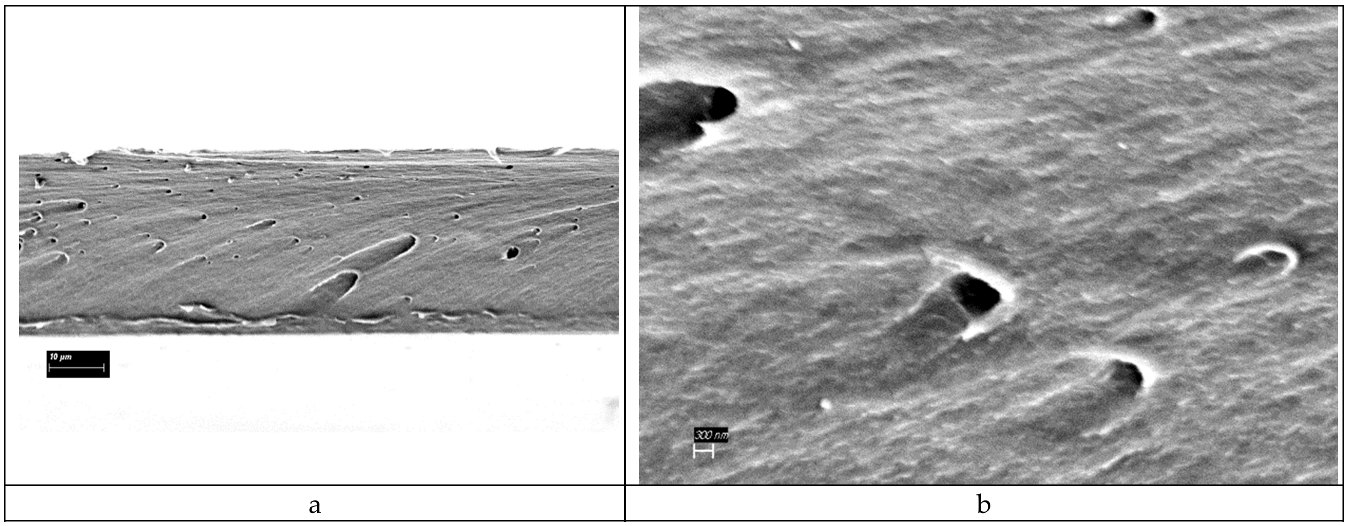

a b

Figure S2. SEM images of the fracture surface ***CoPUIALT900*** film after thermolysis at 350 °C and subsequent alkaline hydrolysis; magnification: ×1000 (a), ×10.000 (b).
